# Impact of an undergraduate dental research training program on evidence-based research outcomes

**DOI:** 10.3389/fdmed.2026.1934334

**Published:** 2026-10-16

**Authors:** Prashen Chelikani, Anastasia Kelekis-Cholakis, Rajinder P. Bhullar

**Affiliations:** 1 Department of Oral Biology, Dr. Gerald Niznick College of Dentistry, University of Manitoba, Winnipeg, MB, Canada; 2 Department of Dental Diagnostics and Surgical Sciences, Dr. Gerald Niznick College of Dentistry, University of Manitoba, Winnipeg, MB, Canada

**Keywords:** dental education, dental student, evidence-based models, research curriculum, research outcomes

## Abstract

**Background:**

Dental schools play a critical role in public health by training oral health professionals to prevent, manage, and treat oral diseases. Academic research is essential to advancing the technologies and knowledge these professionals use to promote public health. More research opportunities are needed to drive progress in and promote oral health research. The University of Manitoba's BSc (Dent) program provides first- or second-year undergraduate dental students opportunities to pursue research while earning a BSc (Dent) degree alongside their Doctor of Dental Medicine (DMD) degree. In 2008, we reported on the general outcomes of this program at the University of Manitoba. In the present study, we aimed to evaluate the research impact of the past 10 years (2016–2025) of the undergraduate BSc (Dent) training program at the University of Manitoba.

**Methods:**

We assessed overall research impact on the current status, academic activities, and achievements of dental students from 2016 to 2025 using information obtained from the Dean's Office of the Dr. Gerald Niznick College of Dentistry at the University of Manitoba. Records were compared to those from 1980 to 2005 at the same university. Citations of peer-reviewed publications were obtained by tracking Google Scholar until September 1, 2026.

**Results:**

In 2016–2025, 104 dental students enrolled in the BSc (Dent) research training program at the University of Manitoba with a cumulative average GPA of 3.65 ± 0.16, compared to 76 trainees enrolled from 1980 to 2005 with a cumulative average GPA of 3.45 ± 0.61. Research metric analysis over this time showed that the 104 DMD students in the BSc (Dent) program submitted or published 64 manuscripts for peer-reviewed publication with 731 total citations, 67 poster presentations, 26 oral presentations, and 16 awards/nominations.

**Conclusion:**

The University of Manitoba trained more dental students in the BSc (Dent) research training program from 2016 to 2025 than from 1980 to 2005. Over the past 10 years, students achieved significant academic milestones, including publishing peer-reviewed articles (37%), delivering poster presentations (37%), oral presentations (15%), and receiving national awards or nominations (9%). These findings suggest that the structured, mentored research experiences provided by the BSc (Dent) program foster critical thinking, which could help these students practice evidence-based dentistry and engage early in academic dentistry and health research.

## 1 Introduction

According to the 2025 statistics from the World Health Organization (https://www.who.int/news-room/fact-sheets/detail/oral-health) (accessed on June 22, 2026), oral diseases, such as periodontal (gum) disease and oral cancer, affect about 3.7 billion people worldwide, posing a significant health and economic burden for many countries and influencing the quality of life of patients by causing discomfort, pain, and even death. Since the first dental school in the world, the Baltimore College of Dental Surgery (Baltimore, Maryland, USA), was established in 1840 (1), millions of oral health care professionals have been trained globally. For example, a total of about 4 million oral health care professionals were recorded worldwide in 2019, including 2.49 million dentists across 192 countries, 1.24 million dental assistants/therapists across 131 countries, and 0.28 million dental prosthetic technicians across 126 countries (2). They significantly contribute to public health in preventing, diagnosing, and treating oral diseases (3).

The Faculty of Dentistry at the University of Toronto is Canada's first dental school, established in 1875 (https://www.dentistry.utoronto.ca/about/history) (accessed on June 22, 2026). This is followed by the establishment of accredited dental schools in nine other universities in Canada, including the University of British Columbia, University of Alberta, University of Saskatchewan, University of Manitoba (UM), Western University, McGill University, Université de Montréal, Université Laval, and Dalhousie University (https://acfd.ca/training-programs/our-schools-programs/).

Advances in technology and knowledge in professional fields, including dentistry, now primarily stem from academic research. Dental students are taught mature, established technologies and knowledge in school for their future professional practice. Training dental students to conduct scientific research and discovery can prepare them for lifelong learning and professional growth, thereby improving patient care by applying research findings and even contributing to these discoveries (4). Furthermore, undergraduate dental basic research education (UDBRE) is critical for cultivating more scientific research capacity in the field (5). However, training undergraduate dental students in research was traditionally (before 1948) not offered in dental schools (6). The creation of the National Institute for Dental Research in the USA in 1948, initiated a focused effort to promote research on oral health problems, and only in 1954 were research efforts in dental schools first funded (6). Since then, more dental schools in the USA and other countries have begun incorporating research into their undergraduate curricula.

The Faculty of Dentistry (now known as the Dr. Gerald Niznick College of Dentistry) at UM was established in 1958 and became the first in Canada to award the Doctor of Dental Medicine (DMD) degree in 1962 (https://www.cda-adc.ca/_files/about/about_cda/history/HSPart4.pdf). It initiated the BSc (Dent) program in 1980 (4), which provides first- or second-year undergraduate dental students with the opportunity to pursue a research project over two summers of their studies. The successful completion of this program leads to the awarding of a BSc (Dent) degree along with the DMD degree. Although many of the 10 accredited dental schools in Canada created similar degrees in the past, UM's BSc (Dent) program is currently the only such program offered in Canada.

Previously, we had evaluated the general outcomes of the BSc (Dent) program at UM over the 25-year period from 1980 to 2005 (4). However, more recent training outcomes of this program have not been systematically reported. This study aimed to evaluate the impact of this research, on knowledge translation and policy, from UM's BSc (Dent) training program over the past 10 years (2016 to 2025). Furthermore, we demonstrate, through the research output, how this program could inform the general public, researchers, and oral health care professionals about advancements in UM's BSc (Dent) program and provide insights into developing strategies to improve BSc (Dent) training outcomes in Canada and other countries.

## 2 Methods

Records of the current status, academic activities, and achievements of BSc (Dent) students from 2016 to 2025 were obtained from the Dean's Office of the Dr. Gerald Niznick College of Dentistry at UM after all individual identifiers had been removed. Over the past 10 years (2016 to 2025), 104 DMD students enrolled in the BSc (Dent) research program at UM. The DMD student intake is 29 students/year. The BSc (Dent) intake varies year by year, averaging 8–10 students/year. Some records were compared with published data for the 25-year period (1980–2005) at UM (4). Citations of peer-reviewed publications were obtained by tracking Google Scholar (https://scholar.google.ca/) until September 1, 2026.

BSc (Dent) students’ data were imported into Microsoft Excel and reviewed for completeness and consistency of category labels. For current status (Figure 1), one record per student was classified as academic, dentist, intern, resident, specialist, student, or unknown; a PivotTable was then used to calculate the frequency and percentage of students in each category, with percentages based on 104 BSc (Dent) students. Scholarly activities (Figure 2) were summarized by counting each reported manuscript for peer-reviewed publication, poster presentation, oral presentation, and award or nomination across all BSc (Dent) students. The percentage for each activity type was calculated using the total number of reported scholarly activities as the denominator (*n* = 171), rather than the number of students. PivotCharts were generated from these summary counts to display the distributions as frequencies and percentages. Images were created in BioRender.

**Figure 1:**
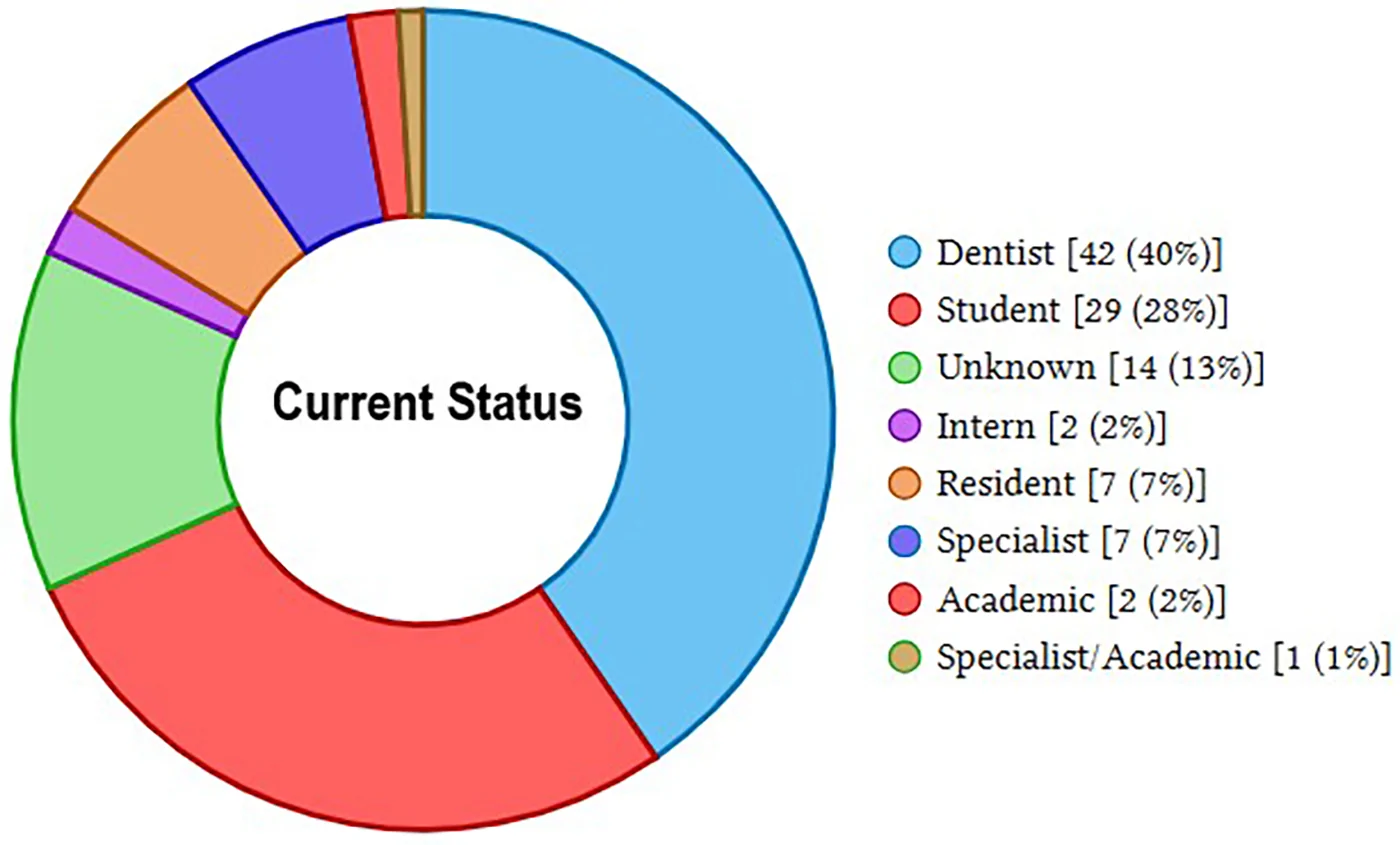
Current status of 104 Doctor of Dental Medicine (DMD) students enrolled in the Bachelor of Science in Dentistry (BSc (Dent)) research training program at the university of Manitoba over the 10 years from 2016 to 2025. Figure was created in BioRender.

**Figure 2:**
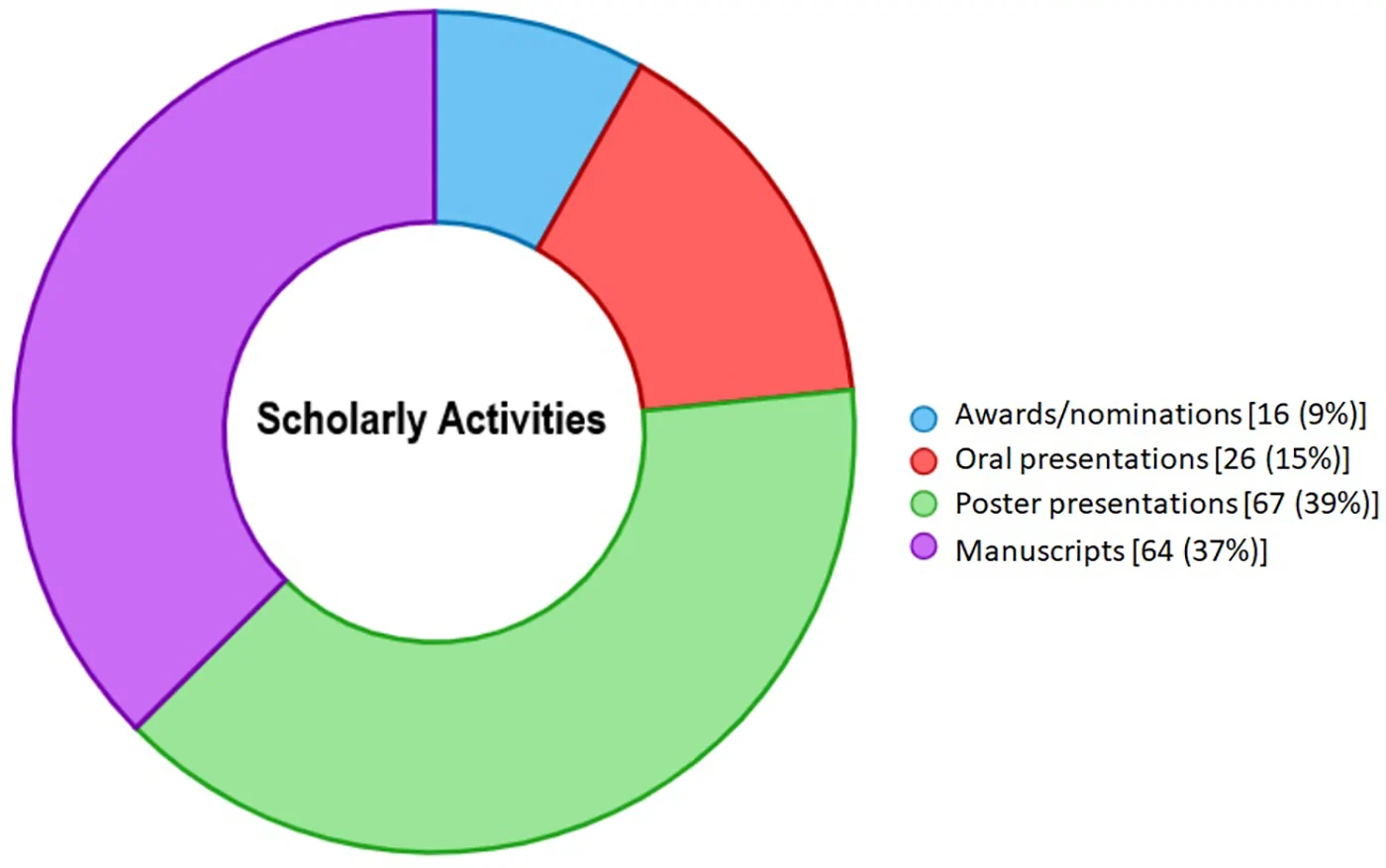
Scholarly activities of 104 Doctor of Dental Medicine (DMD) students enrolled in the Bachelor of Science in Dentistry (BSc (Dent)) research training program at the university of Manitoba over the 10 years from 2016 to 2025. The scholarly activities were classified into four categories: manuscripts submitted for peer-reviewed publication, poster presentations, oral presentations, and national awards/nominations. The manuscripts include 24 manuscripts submitted for peer review and 40 peer-reviewed articles or abstracts accepted or published. Figure was created in BioRender.

BSc Dent and non-BSc Dent students (Tables 1, 2) were compared using a two-sided Welch independent-samples *t*-test, which does not assume equal group variances.

**Table 1:** Comparison of GPAs for admission into the BSc (Dent) program of BSc (Dent) and all DMD students (2012-2024).

Comparison	BSc Dent^a^, mean (SD), *n*	Comparison group, mean (SD), *n*	Mean difference (95% CI)	*t* (df)	*p* value^c^
BSc (Dent)^a^ vs. Class GPA^b^	3.65 (0.16), 104	3.43 (0.10), 354	0.22 (0.187–0.253)	13.28 (127.51)	<0.0001

a Average GPAs of the incoming BSc (Dent) class.

b Average GPAs of all students in the class.

c Two-sided Welch independent-samples t-test.

**Table 2:** Comparison of core course GPA for BSc (Dent) and non-BSc (Dent) DMD students (2012- 2024) at time of admission into the DMD program.

Comparison	BSc Dent^a^, mean (SD), *n*	Comparison group^b^, mean (SD), *n*	Mean difference (95% CI)	*t* (df)	*p* value^c^
BSc (Dent)^a^ vs. non-BSc (Dent)^b^	4.02 (0.14), 104	3.98 (0.07), 250	0.04 (0.012–0.068)	2.77 (124.98)	0.006

a Average GPAs in core required science courses (30 credit hours) at time of admission into the DMD program for students who later enrolled in the BSc (Dent) program.

b Average GPAs for core required science courses (30 credit hours) at time of admission into the DMD program for students who did not pursue the BSc (Dent) program.

c Two-sided Welch independent-samples *t*-test.

## 3 Results

Over the past 10 years (2016 to 2025), 104 DMD students enrolled in the BSc (Dent) research program at UM. Table 1 shows the comparison of the grade point averages (GPAs) for admission into the BSc (Dent) program for BSc (Dent) and all DMD students (includes BSc (Dent) and non-BSc (Dent) students). The cumulative average GPAs for BSc (Dent) and all DMD students were 3.65 ± 0.16 and 3.43 ± 0.10, respectively. Furthermore, the cumulative average GPAs in core science courses (30 credit hours) for BSc (Dent) and non-BSc (Dent) students for admission into the DMD program were 4.02 ± 0.14 and 3.98 ± 0.07, respectively (Table 2). BSc Dent students had a slightly higher mean score than non-BSc Dent students (4.02 versus 3.98), with a mean difference of 0.04 (95% CI: 0.011–0.069; *t* (124.98) = 2.77, *p* = 0.006).

Figure 1 shows the current status of BSc (Dent) students after graduation from the DMD program. Among them, 42 (40%) are dentists, 29 (28%) are current students, two (2%) are engaged in academic research, two (2%) are interns, seven (7%) are residents in clinical postgraduate programs, seven (7%) are specialists, one (1%) is a specialist and is engaged in academic research, and 14 (13%) are of unknown status. No DMD student entered a PhD program and those who entered graduate clinical specialty programs are all research based. Figure 2 shows the outcomes of scholarly activities of 104 BSc (Dent) students, distributed in four categories: manuscripts/abstracts for peer-reviewed publications (37%), poster presentations (39%), oral presentations (15%), and awards/nominations (9%). In more detail, 24 manuscripts have been submitted for peer-reviewed publication, and 40 peer-reviewed articles/abstracts have been accepted or published, with 731 total citations (average 18.3 citations/article) (35% of articles in SJR (SCImago Journal Rank) Q1 journals and 15% of articles in SJR Q2 journals) (Table 3); 67 poster presentations and 26 oral presentations have been delivered, and 16 awards/nominations have been received.

**Table 3:** Information on published articles.

Publication	Article type	Citations^a^	Journal name	SJR ranking	SJR score
(32)	Research	179	J Dent Res	Q1	1.846
(33)	Research		J Anat	Q1	0.694
(21)	Research	20	Nutrients	Q1	1.499
(19)	Research	96	J Dent Res	Q1	1.846
(34)	Research	12	J World Fed Orthod	Q1	1.012
(35)	Research		J World Fed Orthod	Q1	1.012
(36)	Research	36	J Prosthet Dent	Q1	1.291
(37)	Research	4	Cells	Q1	1.69
(7)	Research	9	Front Oral Health	Q1	0.802
(8)	Research	7	Front Oral Health	Q1	0.802
(38)	Research	15	Front Oral Health	Q1	0.802
(39)	Research	4	Front Oral Health	Q1	0.802
(14)	Research	10	Clin. Oral Investig	Q1	0.983
(40)	Research	24	Eur J Orthod	Q1	1.162
(17)	Research		J. Dent. Educ	Q2	0.53
(41)	Research	2	Inorganics	Q2	0.530
(42)	Research	2	J Public Health Dent	Q2	0.58
(43)	Research	1	J Public Health Dent	Q2	0.58
(13)	Research	1	Crystals	Q2	0.497
(12)	Research	10	Bioengineering	Q2	0.811
(44)	Research	1	J Dent Child	Q2/Q3	0.383
(45)	Research	46	J Dent Child	Q2/Q3	0.383
(46)	Research	2	Tanta Dent J	Q3	0.111
(10)	Research	6	Can J Dental Hygiene	Q3	0.433
(47)	Research		J Can Dent Assoc	Q4	0.154
(48)	Research	29	Bone		1.338
(49)	Research	19	J Can Dent Assoc	Q4	0.154
(16)	Research	2	J Can Dent Assoc	Q4	0.154
(11)	Research	43	J Can Dent Assoc	Q4	0.154
(50)	Research	1	Odontostomatol. Trop	Q4	0.108
(51)	Research		J Can Dent Assoc	Q4	0.154
(52)	Research		J Can Dent Assoc	Q4	0.154
(53)	Research		bioRxiv		
(20)	Review	143	Int. J. Biochem. Cell Biol	Q2	0.937
(54)	Methods	1	Methods Microbiol	Q3	2.39
(55)	Book chapter				
(56)	Case report	4	Glob J Oto		
(9)	Report	2			
(57)	Abstract		Int J Oral Maxillofac Surg	Q1	0.79
(58)	Abstract		Eur J Pharm Med Res		
Total citations		731			
Average citations		18.3			

a Google Scholar (Sept 1, 2026). SJR, SCImago Journal Rank.

The BSc (Dent) students received grants and awards from different provincial, national and international agencies. These include nine grants or awards in the range of $2,250 to $7,000 from the Canadian Association of Oral and Maxillofacial Surgeons (CAOMS). The best poster and oral presentations awards from the Manitoba Dental Association (MDA), Canadian Dental Association (CDA) Dentsply Sirona Student Clinician Research Program, the Canadian Association for Dental Research (CADR), International Association for Dental Research (IADR), the Network for Canadian Oral Health Research (NCOHR), Morris award from the American Academy of Oral and Maxillofacial Radiology (AAOMR).

The knowledge translation from these publications is especially noteworthy, as it has the potential to drive changes in policy and dental patient treatment locally and beyond. For example, recent work on examining the impact of the Canada Dental Benefit program (7, 8) may be used to improve the program's implementation or support its ongoing expansion. Furthermore, BSc (Dent) students were involved in the development of the Caries Risk Assessment Tool (9, 10) which can assist non-dental healthcare providers in early detection of caries in non-traditional clinical settings, ultimately enhancing prevention of severe disease cases. Additionally, exploration of prenatal supplementation on future caries risk (11) may help improve prevention strategies for future dental outcomes to ease the health and economic burden of dental caries.

At the clinical level, the research of BSc (Dent) students can help drive clinical practices, such as the recent work investigating the impact of treatment materials and plans (12–15). Even current dental training at the institutional level can be enhanced through research in the BSc (Dent) program, as shown by the evaluation of the geriatric oral health curriculum (16) and factors impacting clinical instructors (17). Contributions of the program to the underlying causes of oral health are also to be included in the impact of the BSc (Dent) program, including microbial contributions to caries (18, 19) and bitter taste biology (20, 21).

These examples demonstrate the wide-ranging impact of the UM BSc (Dent) program, extending from the laboratory bench to evidence-based clinical practice and policy.

## 4 Discussion

In this study, we updated the academic achievements of the BSc (Dent) research program at UM. In the past 10 years (2016–2025), this program has trained and enrolled 104 DMD-BSc (Dent) students, more than the 76 students in the previous 25 years (1980–2005) (4); this represents a greater than a 2.7-fold increase per 10 years. Furthermore, students from the past 10 years have achieved significant academic milestones through publishing peer-reviewed articles, delivering poster and oral presentations, and receiving grants/awards. Given the majority of BSc (Dent) students participated in knowledge translation and scholarly activities and published their scientific findings in peer-reviewed journals (Figure 2 and Table 3) indicates that the BSc (Dent) research program has strengthened their critical thinking, which could help them practice evidence-based dentistry and engage early in academic dentistry and health research.

A study of an 11-year undergraduate dental research program (2007–2017) in China reported that undergraduate dental students who participated in research achieved better GPA scores (3.41 vs. 3.21, *p* < 0.0001), received more awards on the honor rolls (0.53 vs. 0.30 (per student), *p* = 0.0171), and published more peer-reviewed articles (1.62 vs. 1.31 (per student), *p* = 0.0253), compared with those who did not participate in such a program (22). This finding is consistent with the observation in this study that BSc (Dent) students had a higher cumulative average GPA than DMD students in first and second year courses of the DMD program (3.65 ± 0.16 vs. 3.43 ± 0.10) (Table 1). The GPA data, along with the study's findings on awards and peer-reviewed publications, support that research training can improve the academic outcomes of undergraduate dental students. The conclusion is also supported by a survey of 507 dental students and 121 full-time faculty members in Southern California, who reported that 80.6% of dental students and 91.4% of faculty had a positive perspective on the importance of research in dental education, regardless of whether research is mandated or elective (23). A multicenter preliminary study reported insights into the research activities of senior dental students in the Middle East (24). That study surveyed 369 final-year dental students from three institutions. They concluded that research is highly beneficial, especially for improving teamwork and communication skills, as well as data interpretation skills, which positively impacted the research perspectives of 74.1% of students. Based on the feedback received from our BSc (Dent) students and documented in faculty newsletters over the years, the research experience influenced career choice, especially the graduate specialties they wanted to pursue.

Several potential barriers have been identified for developing efficient, effective strategies to improve outcomes in the BSc (Dent) research program in other countries. Many factors can affect the output of the BSc (Dent) research program. A questionnaire-based study conducted in China reported that factors influencing dental undergraduate students’ participation in research include the distribution of scientific resources and a lack of interest or understanding of science (25). Early undergraduate promotion could include distributing scientific resources and enhancing interest and understanding in science (25). To foster dental undergraduate students’ interest in scientific research, dental schools should improve UDBRE by helping students develop scientific questions, design research projects, retrieve and understand literature, write academic articles, conduct experiments, and analyze and evaluate experimental results (26).Other barriers for implementing BSc (Dent) programs which are common to other countries (e.g., China and Pakistan) include, lack of funding, mentorship, and curriculum burden (5, 27, 28).

More strategies designed to overcome the barriers mentioned above may help expand the BSc (Dent) research program to more academic institutions. Strategies to make research more accessible to dental students include developing interesting and relevant research projects, increasing students’ understanding of the importance of research, creating rotating research positions, and adding research components to the basic sciences curriculum during the first two years of dental school (29). Additional approaches include providing extra training, orientation, and time to complete research projects (24). Extracurricular dental basic research education activities (30) and a “flexible” compulsory dental basic research course (31) may also strengthen BSc (Dent) students’ interest and capabilities in scientific research. The growing expectation that clinical postgraduate programs require applicants to demonstrate research experience and output is also driving student interest in the BSc (Dent) program at UM.

Our present study has few limitations. First, our study did not compare training outcomes, in addition to GPAs, between DMD-BSc (Dent) students who participated in research and DMD students who did not. Although every student in the first and second year of the DMD program is eligible to apply, the application process is competitive, and not all applicants are accepted each year. About 20% of first- and second-year DMD students participate in this program. However, given the small class size (29 students), we would estimate that most students interested in research have an opportunity to be accepted into the BSc (Dent) program in the first or second year of their training. Future studies should compare the training outcomes of the BSc (Dent) research program with those of undergraduate dental training programs at other dental schools without a research training component. A second potential limitation is that our study did not investigate qualitative outcomes of the BSc (Dent) research program, such as students’ perceived value of the program, its effect on career progression, and potential barriers encountered.

## 5 Conclusions

In this study, we reported that the BSc (Dent) research program at UM has enrolled more than 2.7 times as many dental students in the past 10 years (2016–2025) as in the previous 25 years (1980–2005). Furthermore, the BSc (Dent) research program in the past 10 years (2016–2025) has achieved academic milestones in peer-reviewed publications (37%), poster presentations (39%), oral presentations (15%), and awards/nominations (8%). The study has limitations, including the omission of a qualitative survey of students on the impact of the BSc (Dent) research program on their career development. The findings of this study support the premise that a BSc (Dent) program is sought after by DMD students at UM, produces credible scholarly activity and research dissemination, and can contribute to evidence-based dental practice and oral public health policy. This study can also help inform the public, researchers, dental professionals, and policymakers about the impact of the BSc (Dent) research program at UM. Future studies can help develop strategies to overcome potential barriers to developing and advancing this type of program across other dental schools, as well as determine the value proposition of such training for students. Future investigations may also explore whether BSc (Dent) programs can directly enhance critical thinking, evidence-based practice, and early engagement in academic dentistry and health research.

## Data Availability

The raw data supporting the conclusions of this article will be made available by the authors, without undue reservation.
